# Expression of PD-1/PD-L1 in peripheral blood and tumor tissues of patients with classical Hodgkin’s lymphoma

**DOI:** 10.1097/MD.0000000000035757

**Published:** 2023-11-03

**Authors:** Xia Feng, Xiangyi Luo, Yingmei Yang, Yuchen Fan, Qing Ye

**Affiliations:** a Department of Integrated Traditional Chinese and Western Medicine, Public Health Clinical Center of Chengdu, Chengdu, China.

**Keywords:** disease staging, Hodgkin’s lymphoma, programmed cell death ligand 1, programmed cell death receptor 1, treatment response assessment

## Abstract

Significant biomarkers can predict and estimate the response to chemotherapy for different types of lymphoma. Classical Hodgkin’s lymphoma (cHL) and peripheral T-cell lymphoma (PTCL) belong to different types of lymphoma, their prognosis is very different, programmed cell death receptor 1 (PD-1) and its ligand (PD-L1) have been studied in these 2 types of diseases. However, few studies have involved the difference in PD-1/PD-L1 levels between cHL and PTCL. To find out the difference and relevant clinical application value, we collected blood samples of 29 newly diagnosed cHL patients and 11 newly diagnosed PTCL ones. At the same time, tumor tissue paraffin sections of 13 patients with cHL were collected at the initial diagnosis. Flow cytometry, enzyme-linked immunosorbent assay, and immunohistochemical staining were used to detect PD-1/PD-L1 levels in peripheral blood T cells, plasma, and tumor tissues, and the relationship between the above results and clinical data of patients in patients with cHL were investigated. The levels of PD-1 on CD4^+^ T cells, PD-L1 on CD4^+^ T cells and PD-1 on CD8^+^ T cells in peripheral blood of cHL and PTCL patients were higher than those of healthy controls, the level of PD-1 in CD4^+^ T cells from peripheral blood was higher from cHL patients with stage III-IV (*P* = .0178), B symptoms (*P* = .0398), higher lactate dehydrogenase (*P* = .0056), higher international prognostic index score (*P* = .0349), and relapsed in later stages (*P* = .0306). The expression level of soluble PD-L1 (sPD-L1) from cHL (*P* < .001) and PTCL (*P* < .0001) patients was higher than that of the healthy control group, and there was higher sPD-L1 level in patients with higher international prognostic index scores (*P* = .0016). The dynamic detection of sPD-L1 showed that after 2 courses of chemotherapy, the sPD-L1 level in cHL patients with complete remission declined, but the level of sPD-L1 from patients with incomplete remission was not significantly changed (*P* > .05). In tumor tissues of cHL patients, PD-1(+) was 77%, PD-L1(+) was 69%, PD-1 and PD-L1 expression levels were high. Our results suggest that PD-1 levels in peripheral blood CD4^+^ T cells are helpful for the stage of disease in patients with cHL, and the dynamic detection of sPD-L1 level is helpful for the judgment of patients with cHL.

## 1. Introduction

Classical Hodgkin lymphoma (cHL) is a kind of B cell-derived malignant lymphoma, accounting for about 20% of all lymphomas. The presence of abundant reactive cells in Reed Sternberg cells and tumor microenvironment is its significant clinicopathological feature, and most Reed Sternberg cells have the expression of programmed cell death ligand 1 (PD-L1), which may be related to tumor cells escaping from the immune response of the body.^[1]^ PD-L1 is highly expressed in melanoma,^[2]^ gastric cancer,^[3]^ liver cancer,^[4]^ ovarian cancer,^[5]^ and other tumors. Previous studies have found that PD-L1, which is highly expressed in tumor cells, interacts with programmed cell death receptor 1 (PD-1) on lymphocytes to inhibit the proliferation and activation of lymphocytes. In this way, tumor cells escape the immune system of the body and survive.^[6]^ However, few studies have been reported on the expression of PD-1 or PD-L1 in peripheral blood from cHL patients. Therefore, we examine the PD-1 or PD-L1 level in 3 types of samples from cHL patients, including T lymphocytes in peripheral blood, plasma, and tumor tissues. We hope to find dynamic monitoring means and efficacy evaluation methods for Hodgkin lymphoma from the way of the PD-1 signaling pathway (immune checkpoints).

## 2. Materials and methods

### 2.1. Study population and treatment

This study included 29 cases of cHL (13 males, median age 25 years) and 11 cases of peripheral T-cell lymphoma (PTCL) diagnosed at West China Hospital of Sichuan University from September 2017 to September 2018. The diagnostic criteria were based on the 2016 WHO classification of hematopoietic and lymphoid tissue tumors.^[7]^ At the time of initial diagnosis, 4 mL EDTA anticoagulant peripheral blood samples were collected from the patients involved, and paraffin samples of tumor tissues from cHL patients were also collected. In addition, 27 healthy volunteers were recruited as healthy controls (13 males, median age 49). During the treatment of cHL patients, peripheral blood samples were collected again after 2 chemotherapy courses. This study has been approved by the Ethics Committee of West China Hospital of Sichuan University (Approval number: No.373) and has signed an informed consent form with all participants.

Twenty-four patients (86%) received the ABVD chemotherapy scheme, and radiotherapy was considered after at least 2 courses of chemotherapy. Therefore, the efficacy evaluation results after 2 courses of chemotherapy were not affected by radiotherapy. The therapeutic effect was determined according to the Evaluation Criteria for the Efficacy of Malignant Lymphoma, including complete remission (CR), partial remission, stable disease, and disease progression.^[8]^ The results showed that 18 patients (62%) reached CR, and the remaining 11 patients (38%) did not reach CR (non-CR, including 10 partial remission and 1 disease progression).

### 2.2. Main reagents and instruments

FITC anti human CD279 (PD-1) (Catlog: 329904, CA), PE Anti Human CD274 (PD-L1, B7-H1) (Catlog: 4307499) fluorescent antibodies were purchased from eBioscience, APC anti human CD4 (Catlog: H10041-11I, Tianjin, China), PE/Cy7 anti-human CD8 (Catalog: H20081-17H) fluorescent antibodies were purchased from Tianjin Sanjian Biological Co., Ltd. Red cell lysate was purchased from Beijing Leigen Biotechnology Co., Ltd., and multi-color flow cytometer was purchased from American Beckman Company. The human peripheral blood lymphocyte isolation solution was purchased from Tianjin Haoyang Biological Co., Ltd. The soluble PD-L1 (sPD-L1) enzyme-linked immunosorbent assay (ELISA) kit was purchased from American Abcam Company, the mouse anti-human PD-1 antibody (primary antibody) was purchased from Beijing Zhongshan Jinqiao Company, the rabbit anti-human PD-L1 [28-8] antibody (primary antibody) (catalog: ab277712, Cambridge, UK) was purchased from American Abcam Company, and the anti rabbit/mouse (secondary antibody) (Envision^™^ Detection Kit (catalog: K5007, Copenhagen, Denmark)) universal immunohistochemistry test kit was purchased from Danish DAKO Company.

### 2.3. Method

#### 2.3.1. Flow cytometry analysis.

Five hundred microliters of peripheral blood from patients was treated with ACK red blood cells lysis solution (Leakene, Beijing, China), washed twice with phosphate-buffered saline (PBS), and then incubated in the dark for 15 minutes (eBioscience, CA) with antibodies targeting CD4 (conjugated with fluorescent group APC), CD8 (PE-Cy7), PD-1 (FITC), or PD-L1 (PE). The samples were washed twice with PBS, resuspended in 300 μL PBS, and detected by flow cytometry (Navio Beckman Coulter, CA). Finally, the data was analyzed by FlowJo X 10 software.

#### 2.3.2. ELISA of soluble PD-L1 in plasma.

The plasma sample was used to quantify the plasma sPD-L1 concentration through the human PD-L1 [28-8] ELISA kit (catalog no. 28-8, Abcam, Cambridge, UK) according to the instructions of the reagent kit. The minimum detectable concentration of this kit is 2.91 pg/mL.

#### 2.3.3. Immunohistochemical staining of tumor tissue paraffin sections.

Paraffin sections (3 μm) of tumor tissue were used for immunohistochemical staining as previously described.^[9]^ The primary antibody (PD-1 or PD-L1, dilution 1:75) and universal secondary antibody (Envision™ Detection Kit(Code No: K5007)) were sequentially added for antibody incubation. The positive rate of PD-1 and PD-L1 expression (number of positive cells/number of all cells) was analyzed by Image J software. The positive rate >30% was defined as PD-L1 positive, and the positive rate >5% was PD-1 positive. This positive standard was based on the literature report.^[10]^

### 2.4. Statistical analysis

GraphPad Prism 8.0 software was used for statistical analysis of the data. The test results are expressed by median and interquartile intervals [median (interquartile range)]. Kruskal–Wallis test is used to analyze the differences between groups, Mann–Whitney *U* test and Spearman rank correlation analysis are used to analyzing the relationship between the test results and the clinical data of patients, Wilcoxon paired sign rank test is used to compare the changes of SPD-L1 levels of the same patient at the time of diagnosis and the end of 2 courses of treatment. Set *P* value < .05 as the difference is statistically significant.

## 3. Results

### 3.1. Baseline clinical characteristics of patients

The baseline clinical characteristics of cHL or PTCL patients are summarized in Table 1 (The clinical data of patients were from the medical records and examination data of patients in Sichuan University). 13 patients (45%) in stage III-IV, 8 patients (28%) with lactate dehydrogenase (LDH) > 250 U/L. All patients are considered for radiation therapy after receiving at least 2 courses of chemotherapy. After 2 courses of chemotherapy, 18 patients (62%) achieved CR. We also recruited 27 healthy volunteers (13 males and 14 females) as a control group, with a median age of 49 years (interquartile range, 34–55) (data not shown).

**Table 1:** Baseline and clinical characteristics of 40 patients.

Characteristics	cHL patients (n = 29)	PTCL patients (n = 11)
Age, yr		
>60	4 (14)	6 (55)
≤60	25 (86)	5 (45)
Sex		
Male	13 (45)	9 (82)
Female	16 (55)	2 (18)
Ann Arbor stage		
I–II	16 (55)	3 (27)
III–IV	13 (45)	8 (73)
Bulky mass		
Yes	18 (62)	6 (55)
No	11 (38)	5 (45)
B symptoms		
Yes	8 (28)	5 (45)
No	21 (72)	6 (55)
Extranodal sites		
>1	9 (31)	7 (64)
≤1	20 (69)	4 (36)
ECOG PS		
≥1	9 (31)	5 (45)
<1	20 (69)	6 (55)
Lactate dehydrogenase (U/L)		
>250	8 (28)	7 (64)
≤250	21 (72)	4 (36)
EBER(+)		
Yes	7 (24)	5 (45)
No	22 (76)	6 (55)
IPI		
Low (0–2)	25 (86)	5 (45)
High (≥3)	4 (14)	6 (55)
Response after 2 treatment courses		
Complete remission	18 (62)	1 (9)
Other	11 (38)	10 (91)
Relapse		
Yes	8 (28)	-
No	21 (72)	-

Values are n (%).

“-” = data not collected, cHL = classical Hodgkin lymphoma, EBER = Epstein–Barr encoded RNA, ECOG PS = Eastern Cooperative Oncology Group performance score, IPI = international prognostic index, PTCL = peripheral T-cell lymphoma.

### 3.2. Expression of PD-1 or PD-L1 in peripheral blood T cells of lymphoma patients

**Figure 1.**
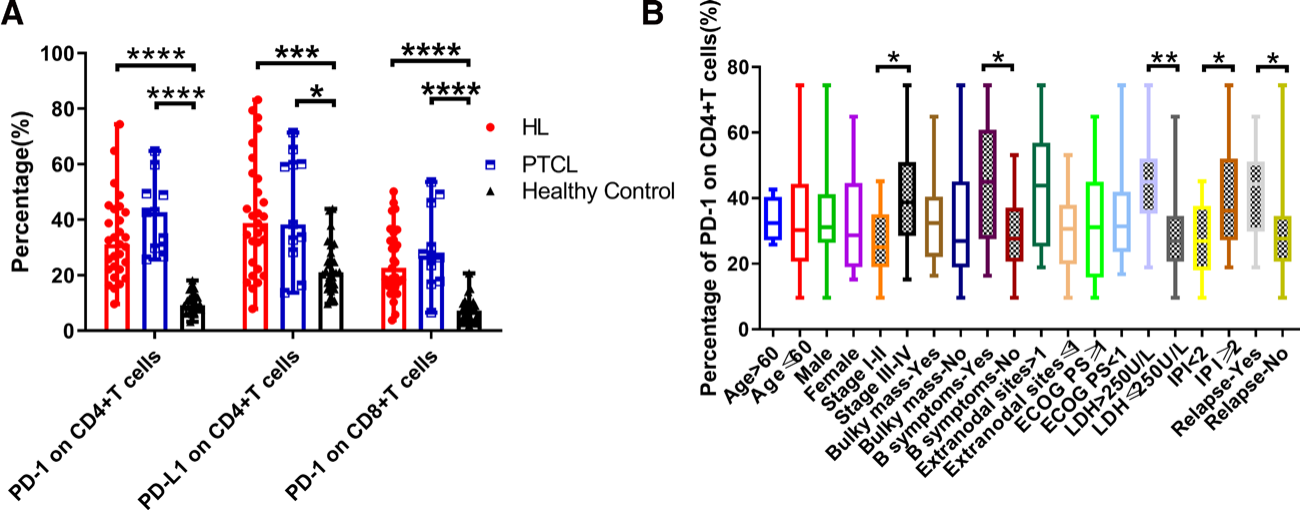
(A) Positive percentages of CD4^+^ or CD8^+^ T cells expressing PD-1 or PD-L1 from cHL or PTCL or healthy volunteers. (B) Correlation of PD-1 level of CD4^+^ T cells in cHL patients with disease stage, B symptoms, LDH, and IPI. ****P < .0001, ***P < .001, **P < .01, and *P < .05. cHL = classical Hodgkin’s lymphoma, IPI = international prognostic index, LDH = lactate dehydrogenase, PD-1 = programmed cell death receptor 1, PD-L1 = programmed cell death ligand 1, PTCL = peripheral T-cell lymphoma.

The results of flow cytometry showed that the expression levels of CD4^+^ T cell PD-1, CD4^+^ T cell PD-L1, and CD8^+^ T cell PD-1 in peripheral blood of cHL and PTCL patients were higher than those of the healthy control group (Fig. 1A, Table 2). The analysis results of the relationship between flow cytometry results and clinical characteristics showed that the level of PD-1 in CD4^+^ T cells from peripheral blood was higher in cHL patients with stage III-IV (*P* = .0178), B symptoms (*P* = .0398), higher LDH (*P* = .0056), higher international prognostic index (IPI) score (*P* = .0349), and relapsed in later stages (*P* = .0306) (Fig. 1B). In addition, Spearman rank correlation analysis found that the level of PD-L1 on CD4^+^ T cells was negatively correlated with the absolute lymphocyte count (Spearman *r* = −0.5997, *P* = .0323, Fig. 1B).

**Table 2:** Expression of PD-1/PD-L1 on T lymphocytes in peripheral blood of lymphoma patients and healthy volunteers.

Group	N	PD-1 level on CD4^+^ T cell (%), median (IQR)	*P*	PD-L1 level on CD4^+^ T cell (%), median (IQR)	*P*	PD-1 level on CD8^+^ T cell (%), median (IQR)	*P*
cHL	29	31.10 (22.10–43.20)	<.0001	38.70 (23.40–55.75)	<.001	22.60 (15.95–34.00)	<.0001
PTCL	11	42.60 (29.70–49.40)	<.0001	38.30 (28.30–60.00)	<.05	27.00 (17.80–46.10)	<.0001
Healthy control	27	9.20 (7.70–12.40)	–	21.10 (16.80–28.10)	–	7.20 (4.20–9.80)	–

Values are median (IQR).

cHL = classical Hodgkin lymphoma, IQR = interquartile range, PD-1 = programmed cell death receptor 1, PD-L1 = programmed cell death ligand 1, PTCL = peripheral T-cell lymphoma.

### 3.3. Expression of sPD-L1 in plasma of lymphoma patients and healthy volunteers

**Figure 2.**
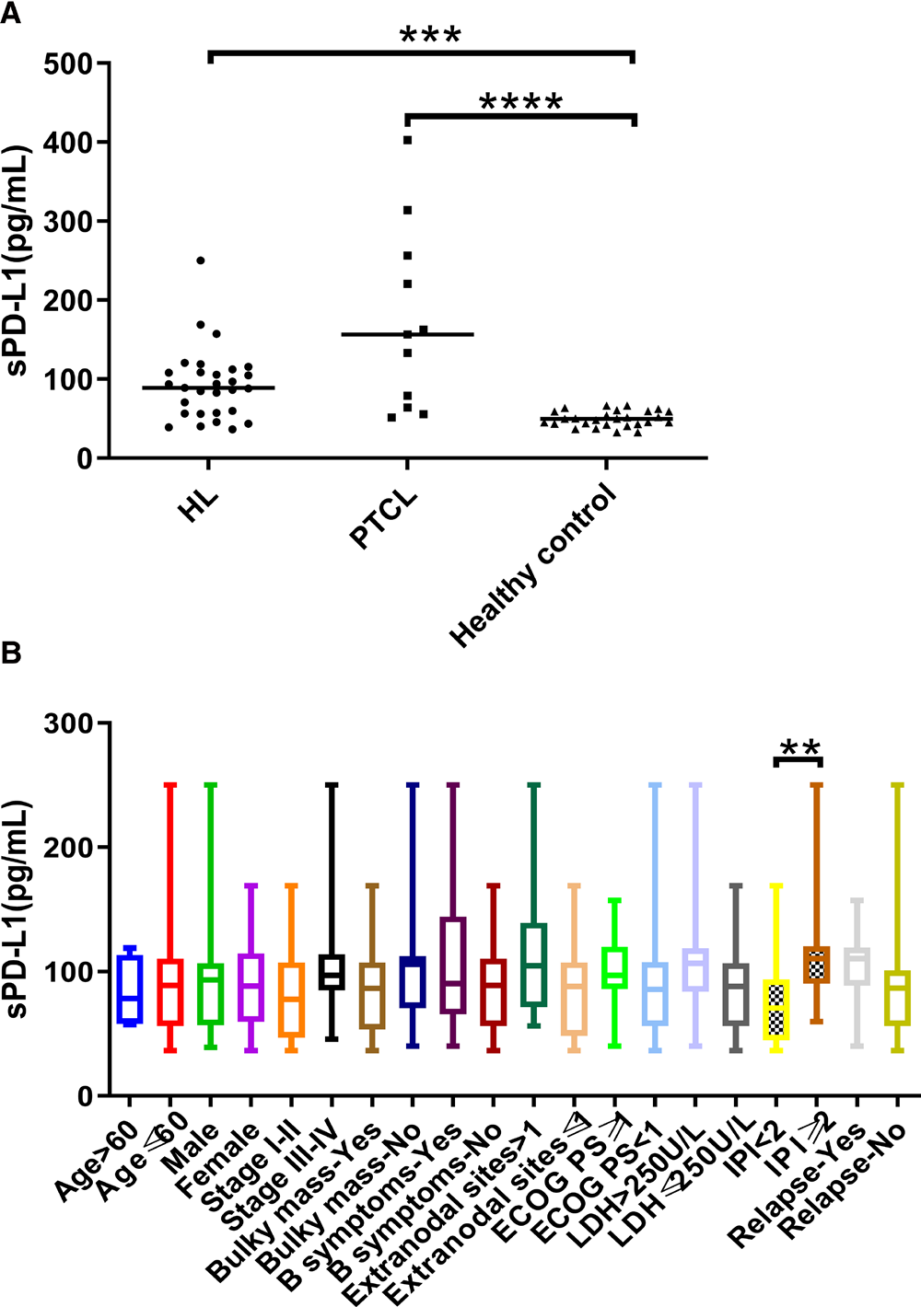
(A) The expression of sPD-L1 in plasma of lymphoma patients and healthy volunteers. (B) The relationship between sPD-L1 level and clinical characteristics in patients with cHL. ***P < .001, ****P < .001, and **P < .01. cHL = classical Hodgkin’s lymphoma, sPD-L1 = soluble PD-L1.

ELISA results showed that the expression level of sPD-L1 in plasma of cHL (*P* < .001) and PTCL (*P* < .0001) patients was higher than that of the healthy control group (Fig. 2A), and there was higher sPD-L1 level in patients with higher IPI scores (*P* = .0016) (Fig. 2B).

### 3.4. Efficacy evaluation of cHL patients

**Figure 3.**
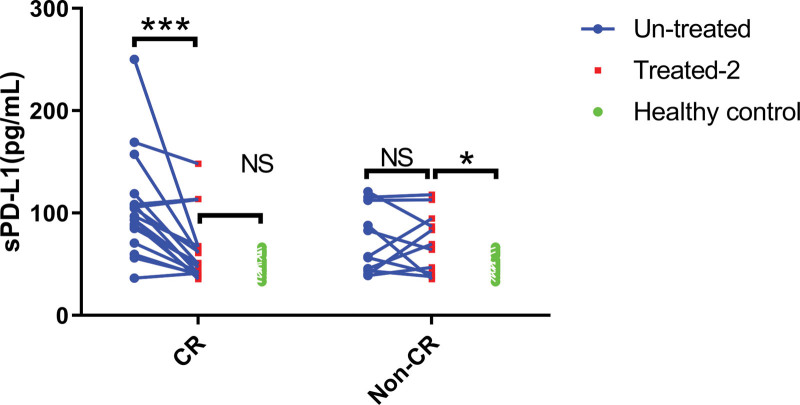
The relationship between the expression level of sPD-L1 and the therapeutic effect in cHL patients. ***P < .001 and *P < .05. cHL = classical Hodgkin’s lymphoma, sPD-L1 = soluble PD-L1.

The dynamic detection and analysis results of sPD-L1 are shown in Figure 3. In the CR group, after 2 courses of chemotherapy, the level of sPD-L1 dropped to the level of the healthy control group. In the non-CR group, the sPD-L1 level after chemotherapy was not significantly different from that before chemotherapy and was higher than that of the healthy control group.

### 3.5. Expression of PD-1/PD-L1 in tumor tissues of cHL patients

**Figure 4.**
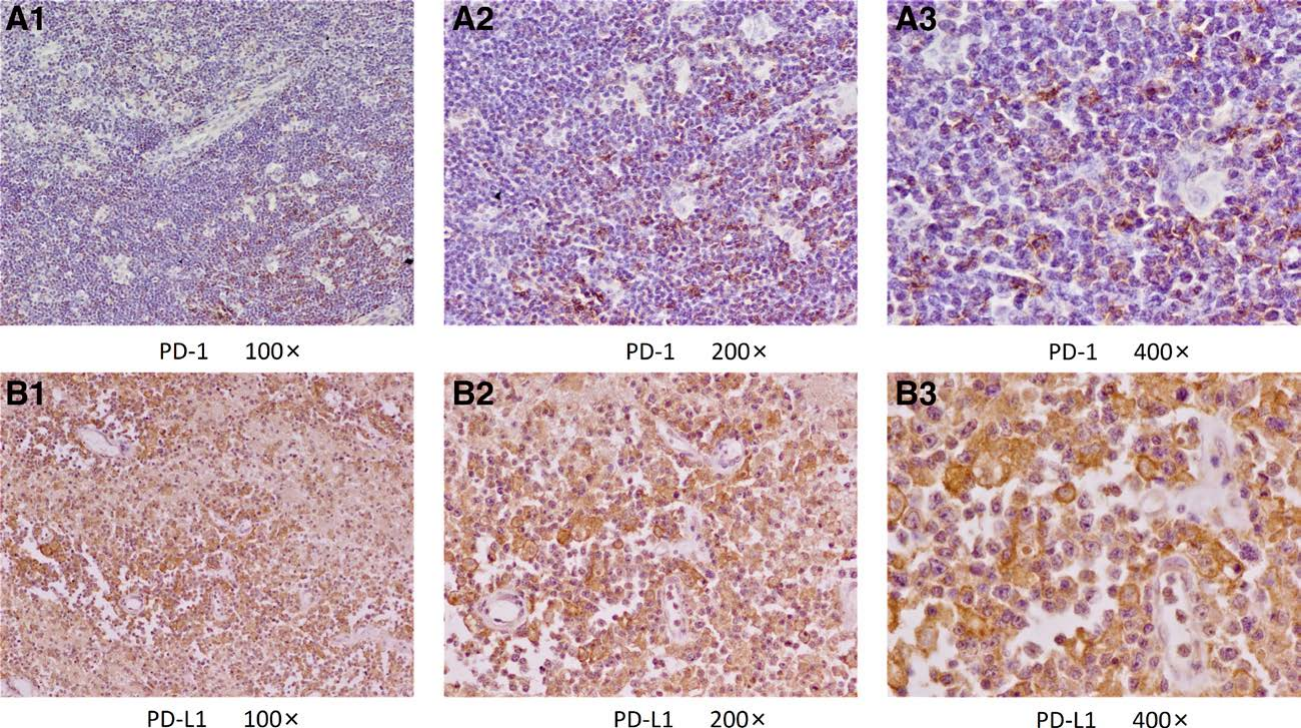
PD-1 or PD-L1 immunohistochemical staining in tumor tissue of a typical cHL patient. cHL = classical Hodgkin’s lymphoma, PD-1 = programmed cell death receptor 1, PD-L1 = programmed cell death ligand 1.

Immunohistochemical staining of PD-1 or PD-L1 was performed on paraffin sections of tumor tissues of 13 cHL patients. The distribution and expression of PD-1/PD-L1 in tumor tissues of a typical patient are shown in Figure 4. The membrane of positive cells was stained brown. PD-L1 (+) was defined as the proportion of positive cells >30%, and PD-1 (+) was defined as the proportion of positive cells >5%; PD-1 (+) in tumor tissues of cHL patients was 10 cases (77%); 9 cases of PD-L1 (+), accounting for 69%

### 3.6. Relationship between PD-1 or PD-L1 level of peripheral blood T lymphocytes, sPD-L1 level and PD-1/PD-L1 level in tumor tissues of cHL patients

Spearman rank correlation analysis showed that the levels of PD-1/PD-L1 in peripheral blood T lymphocytes, sPD-L1, and PD-1/PD-L1 in tumor tissues of cHL patients were not statistically significant (data not shown).

## 4. Discussion

Previous studies have confirmed that the PD-1/PD-L1 signaling pathway plays an important role in the regulation of the peripheral blood immune system in various tumor patients. We have confirmed that PD-1 level is significantly increased in CD4^+^ T cells or CD8^+^ T cells from peripheral blood in solid tumors such as non-small cell lung cancer,^[11]^ oral squamous cell carcinoma,^[12]^ and ovarian cancer.^[13]^ Similarly, we also found that the expression levels of PD-1 on CD4^+^ or CD8^+^ T cells and PD-L1 on CD4^+^ T cells from peripheral blood from cHL patients are higher than those in the healthy control group.

Other studies have shown that the level of PD-1 in peripheral blood T lymphocytes is positively correlated with the staging and clinical progression of cervical cancer^[14]^ and gastric cancer.^[15]^ Similarly, this study also found that PD-1 levels on CD4^+^ T cells from peripheral blood was higher in cHL patients with stage III-IV, B symptoms, higher IPI score, and higher LDH concentration. This implied that PD-1 level on CD4^+^ T cells from peripheral blood contributes to the staging of cHL patients. In addition, PD-L1 is mainly overexpressed on tumor cells, but this study found that the expression of PD-L1 on CD4^+^ T cells from peripheral blood in cHL patients was up-regulated. It has been reported that PD-L1 is up-regulated on CD4^+^ regulatory T cells in Hodgkin lymphoma tissue and inhibits the function of PD-1 (+) T cells.^[16]^ Therefore, we speculated that a high level of PD-L1 from CD4^+^ T cells can inhibit the function of the PD-1 (+) T cells by binding with PD-1 on other T cells, to reduce the function of the peripheral blood immune system and promote tumor immune escape. In addition, sPD-L1 levels in cHL patients decreased to healthy control levels after achieving complete remission through chemotherapy, indicating that dynamic monitoring of sPD-L1 can be used for efficacy evaluation. Finally, the immunohistochemical staining results in the tumor tissue of cHL patients showed that the positive rates of PD-1 and PD-L1 were higher in the tumor tissue of cHL patients, which may be one of the factors contributing to the better efficacy of PD-1 monoclonal antibodies in HL patients.

In summary, the results of this study show that the expression levels of PD-1 on peripheral blood CD4^+^ T cells are helpful for disease staging in cHL patients, while dynamic detection of sPD-L1 helps evaluate the efficacy of cHL patients. However, the sample size of this study is not sufficient, the duration of the study is relatively short, and sufficient follow-up of patients has not been conducted. Further expansion of the sample size and extension of follow-up time should be conducted to verify this.

## Author contributions

**Data curation:** Xia Feng, Yingmei Yang, Yuchen Fan.

**Formal analysis:** Xia Feng, Xiangyi Luo, Yuchen Fan.

**Funding acquisition:** Qing Ye.

**Investigation:** Xia Feng, Qing Ye.

**Project administration:** Qing Ye.

**Validation:** Xia Feng.

**Visualization:** Xiangyi Luo.

**Writing – original draft:** Xia Feng.

**Writing – review & editing:** Xia Feng, Yuchen Fan.

## References

[1] Nagato T Ohkuri T Ohara K. Programmed death-ligand 1 and its soluble form are highly expressed in nasal natural killer/T-cell lymphoma: a potential rationale for immunotherapy. Cancer Immunol Immunother. 2017;66:877–90. 28349165 10.1007/s00262-017-1987-x PMC11028583

[2] Chen G Huang AC Zhang W. Exosomal PD-L1 contributes to immunosuppression and is associated with anti-PD-1 response. Nature. 2018;560:382–6. 30089911 10.1038/s41586-018-0392-8 PMC6095740

[3] Wu C Zhu Y Jiang J. Immunohistochemical localization of programmed death-1 ligand-1 (PD-L1) in gastric carcinoma and its clinical significance. Acta Histochem. 2006;108:19–24. 16530813 10.1016/j.acthis.2006.01.003

[4] Gao Q Wang XY Qiu SJ. Overexpression of PD-L1 significantly associated with tumor aggressiveness and postoperative recurrence in human hepatocellular carcinoma. Clin Cancer Res. 2009;15:971–9. 19188168 10.1158/1078-0432.CCR-08-1608

[5] Abiko K Matsumura N Hamanishi J. IFN-gamma from lymphocytes induces PD-L1 expression and promotes the progression of ovarian cancer. Br J Cancer. 2015;112:1501–9. 25867264 10.1038/bjc.2015.101 PMC4453666

[6] Okazaki T Maeda A Nishimura H. PD-1 immunoreceptor inhibits B cell receptor-mediated signaling by recruiting src homology 2-domain-containing tyrosine phosphatase 2 to phosphotyrosine. Proc Natl Acad Sci USA. 2001;98:13866–71. 11698646 10.1073/pnas.231486598 PMC61133

[7] Sabattini E Bacci F Sagramoso C. WHO classification of tumors of hematopoietic and lymphoid tissues in 2008: an overview. Pathologica. 2010;102:83–7. 21171509

[8] Cheson BD Pfistner B Juweid ME.; International Harmonization Project on Lymphoma. Revised response criteria for malignant lymphoma. J Clin Oncol. 2007;25:579–86. 17242396 10.1200/JCO.2006.09.2403

[9] Otali D Fredenburgh J Oelschlager DK. A standard tissue as a control for histochemical and immunohistochemical staining. Biotech Histochem. 2016;91:309–26. 27149658 10.1080/10520295.2016.1179342 PMC5338041

[10] Kiyasu J Miyoshi H Hirata A. Expression of programmed cell death ligand 1 is associated with poor overall survival in patients with diffuse large B-cell lymphoma. Blood. 2015;126:2193–201. 26239088 10.1182/blood-2015-02-629600 PMC4635115

[11] Waki K Yamada T Yoshiyama K. PD-1 expression on peripheral blood T-cell subsets correlates with prognosis in non-small cell lung cancer. Cancer Sci. 2014;105:1229–35. 25117757 10.1111/cas.12502 PMC4462362

[12] Zhang P Ouyang S Wang J. [Levels of programmed death-1 and programmed death ligand-1 in the peripheral blood of patients with oral squamous cell carcinoma and its clinical implications]. Hua xi kou qiang yi xue za zhi. 2015;33:529–33. 26688950 10.7518/hxkq.2015.05.019 PMC7030333

[13] Maine CJ Aziz NH Chatterjee J. Programmed death ligand-1 over-expression correlates with malignancy and contributes to immune regulation in ovarian cancer. Cancer Immunol Immunother. 2014;63:215–24. 24297569 10.1007/s00262-013-1503-x PMC11029577

[14] Zhang Y Zhu W Zhang X. Expression and clinical significance of programmed death-1 on lymphocytes and programmed death ligand-1 on monocytes in the peripheral blood of patients with cervical cancer. Oncol Lett. 2017;14:7225–31. 29344157 10.3892/ol.2017.7105 PMC5754902

[15] Saito H Kuroda H Matsunaga T. Increased PD-1 expression on CD4+ and CD8+ T cells is involved in immune evasion in gastric cancer. J Surg Oncol. 2013;107:517–22. 23129549 10.1002/jso.23281

[16] Yang ZZ Novak AJ Stenson MJ. Intratumoral CD4+CD25+ regulatory T-cell-mediated suppression of infiltrating CD4+ T cells in B-cell non-Hodgkin lymphoma. Blood. 2006;107:3639–46. 16403912 10.1182/blood-2005-08-3376 PMC1895773

